# The EU Elephant: Europe in the 2021 Dutch General Elections

**DOI:** 10.1007/s10272-021-0956-y

**Published:** 2021-04-06

**Authors:** Simon Otjes

**Affiliations:** Political Science, Leiden University, Rapenburg 70, 2311 EZ Leiden, Netherlands

## Abstract

For the Netherlands, the single most important EU issue is the future of the eurozone.

The Netherlands held general elections in March 2021, the first in a series of national elections in the Netherlands, Germany, France and Hungary that could determine the course of the European Union in the coming years. As had often been the case in the past, televised debates were the most important arena where the Dutch election campaign was fought. EU integration did not play a major role in the campaign and was only mentioned in a single debate on the day before the elections. The absence of a serious debate about the European Union led a group of academics and political observers to introduce the “EU Elephant” in the public debate (Boekestijn, 2021; Boekestijn et al., 2021; Groen, 2021). In their view, the EU was the elephant in the room that parties did not address in the campaign. Even when parties address the issue in public, the Dutch debate often flattens into a pro-/anti-EU debate. This does not reflect that the Netherlands – the largest of the small member states – may likely shape what the EU will look like.

While the EU was absent from the debate in the run-up to the elections, it still appears to have played some role in the outcome, showing a country that is deeply conflicted about the future of the Union. Moreover, the results of the elections and – perhaps more important in the Dutch case – the ensuing cabinet formation, will determine the shape of the future cabinet’s EU policy. In the last 11 years, the Netherlands, under the leadership of Liberal Prime Minister Mark Rutte, has certainly been a brake on any step that would transform the EU into a transfer union between economically stronger Northern European countries and economically weaker Southern countries.

This article discusses the election campaign, the result of the elections, the balance between pro-European and Eurosceptic parties and which policies the current Dutch government is likely to pursue.

**Simon Otjes**, Leiden University; and Groningen University, Netherlands.

## An expected victor and an unexpected challenger

Due to the coronavirus crisis, traditional campaigning through canvassing and physical events was not possible for most parties.[1] The campaign was mainly waged via traditional media, in particular through three televised debates, and social media. The key issue in the campaign was leadership. The Liberal Party, VVD, focused their campaign on Prime Minister Mark Rutte. He has been in power since 2010 and is thus one of the longest sitting prime ministers in Europe. Dutch voters believed that Rutte was the most capable leader to steer the country through the COVID-19 crisis. There had been a sharp increase in trust in Rutte in spring 2020 in what researchers have called a “rally ‘round the flag effect” (Van der Meer et al., 2020). A year before the elections, some observers already claimed that he was unbeatable (Eijsden, 2020). This increased appreciation of Rutte persisted until the elections, although in the final months, as the political debate heated up again, there was a dip in his support (Kanne and Driessen, 2021).

The alternative to Rutte’s leadership came from two of his own ministers, one of whom was Sigrid Kaag, the Minister of Foreign Trade and Development Cooperation and the newly elected leader of the social liberal Democrats 66. Kaag promised “new leadership” for the Netherlands based on her progressive, pro-European and cosmopolitan values. The multilingual former diplomat caught the imagination of the Dutch public when she had briefly served as the Minister of Foreign Affairs when VVD Minister Halbe Zijlstra had to step down early in the cabinet’s term. The realistic possibility of having a female prime minister made her attractive to progressive voters.

The second alternative was Wopke Hoekstra, the Minister of Finance. He was officially appointed to lead the Christian Democratic Appeal (CDA) just two months before the elections. He replaced the previous leader of the CDA, Health Minister Hugo de Jonge, who had to step down because it was not possible to lead the CDA while simultaneously serving as the main minister dealing with the COVID-19 crisis. Hoekstra said he would “press ahead now” under his leadership, while at the same time promising a break with Rutte’s liberal policies. Hoekstra’s harsh negotiating stance on the European Multiannual Financial

1 The exception was FVD, which denied the severity of COVID-19 and held rallies in many Dutch cities.

Table 1
**Seat distribution in 2017 and 2021**

| Abb. | Name Dutch | Name English | Leader | G/O[a] | Ideology | EU position | 2017 | 2021 |
|---|---|---|---|---|---|---|---|---|
| VVD | Volkspartij voor Vrijheid en Democratie | Liberal Party | Mark Rutte | G | Conservative liberal | Euro-pragmatist | 33 | 34 |
| D66 | Democraten 66 | Democrats 66 | Sigrid Kaag | G | Social liberal | Euro-federalist | 19 | 24 |
| PVV | Partij voor de Vrijheid | Freedom Party | Geert Wilders | O | Radical right-wing populist | Hard Eurosceptic | 20 | 17 |
| CDA | Christen-Democratisch Appèl | Christian Demo-cratic Appeal | Wopke Hoekstra | G | Christian democratic | Euro-pragmatist | 19 | 15 |
| SP | Socialistische Partij | Socialist Party | Lilian Marijnissen | O | Socialist | Soft Eurosceptic | 14 | 9 |
| PvdA | Partij van de Arbeid | Labour Party | Lilianne Ploumen | O | Social democratic | Pro-European | 9 | 9 |
| GL | GroenLinks | GreenLeft | Jesse Klaver | O | New left | Pro-European | 14 | 8 |
| FVD | Forum voor Democratie | Forum for Democracy | Thierry Baudet | O | Radical right-wing populist | Hard Eurosceptic | 2 | 8 |
| PvdD | Partij voor de Dieren | Party for the Animals | Esther Ouwehand | O | Deep green | Soft Eurosceptic | 5 | 6 |
| CU | ChristenUnie | ChristianUnion | Gert-Jan Segers | G | Christian social | Euro-pragmatist | 5 | 5 |
| Volt | | | Laurens Dassen | O | Euro-federalist | Euro-federalist | - | 3 |
| JA21 | | Yes21 | Joost Eerdmans | O | Radical right-wing populist | Soft Eurosceptic | - | 3 |
| SGP | Staatkundig Gereformeerde Partij | Reformed Political Party | Kees van der Staaij | O | Christian conservative | Soft Eurosceptic | 3 | 3 |
| DENK | | Think/Equal[b] | Farid Azarkan | O | Multiculturalist | Euro-pragmatist | 3 | 3 |
| 50PLUS | | | | O | Pensioners' interest | Euro-pragmatist | 4 | 1 |
| BBB | BoerBurgerBeweging | Farmer-Citizen Movement | Caroline van der Plas | O | Agrarian interest | Soft Eurosceptic | - | 1 |
| BIJ1 | | As1 | Sylvana Simons | O | Intersectional feminist | Pro-European | 0 | 1 |

Notes: [a] G = Government, O = Opposition; [b] Denk means "think" in Dutch and "equal" in Turkish.

Source: Author's compilation.

Framework may not have been received well outside of the Netherlands (De Witt Wijnen and Van Wiel, 2020), but it was received well at home and made him a potential successor to Rutte.

In the televised debates, Rutte was able to keep the opposition at bay, but Kaag's star rose while Hoekstra's declined (Kester, 2021). Hoekstra was unfamiliar with the details of his own programme, which included harsh cuts to social security. Kaag showed her skills in the debates, in particular after she was attacked by Geert Wilders of the Freedom Party for wearing a headscarf when visiting the Iranian government in her capacity as acting Minister of Foreign Affairs. She strongly denied Wilders's accusation that she had committed treason by doing so, noting instead that she had acted in the country's interest by advocating for peace and stability in the region. The perspective of 'new leadership' under Kaag became a real possibility in the waning days of the campaign.

## Victors and losers

The new old and new composition of the Dutch *Tweede Kamer*, as well as all 17 parties currently in parliament, are listed in Table 1. D66 won five seats more than in 2017. Kaag ended up winning 24 out of 150 seats (as much as the party's legendary leader Hans van Mierlo did in 1994). This was an exceptional feat as governing had always cost D66 dearly in the elections. The party drew support from the pro-European progressive parties: The progressive left-wing GreenLeft lost six out 14 seats, while the social democratic Labour Party kept nine seats but lost all the gains it had made in the polls after its disastrous 2017 loss. At the pro-European side of the spectrum, a new party also entered parliament: Volt. This party has a social liberal manifesto and is committed to European federalism. The party presents itself as the Dutch branch of a pan-European party that also has a single (German) representative in the European Parliament.

The VVD ended up winning just one more seat than its 2017 total. Still, the liberals were the largest party in parliament. The CDA lost five seats (falling from 19 to 14) and the fourth government party, the Christian social CU, kept its five seats. All in all, the government parties expanded their seat total compared to 2017. It is the first time since 1998 that the parties supporting the government actually expanded their seats, and the first time since 2003 that the government parties won a majority.

We also saw notable shifts in seats for the Eurosceptic parties. The Dutch *Tweede Kamer* currently has three radical right-wing populist partiess, which all combine some degree of nativism, authoritarianism, populism and Euroscepticism. The largest, the Freedom Party of Geert Wilders, lost three of its 20 seats. It advocates a Nexit and focuses its nativism on Islam. The Forum for Democracy of Thierry Baudet expanded its support from two to eight. Half a year before the elections, the party had become embroiled in a scandal about anti-Semitic memes shared by members of its youth organisation. The moderate wing left the party to form a new party, Yes21. Baudet retained the leadership of his party and refocused its course on COVID-19. The party focused its entire campaign on ending the lockdown measures. They argued that the coronavirus crisis was overstated, and that the lockdown measures could be lifted as long as the vulnerable were isolated. It no longer focused on its Eurosceptic and anti-immigration manifesto. Yes21 is more moderate also in its Euroscepticism, not advocating a Dutch exit from the EU but merely proposing to bring the EU back to a free trade association. This party won three seats. Together the radical right-wing populists won 28 seats, as much as the right-wing populist parties had done in 2002 under Pim Fortuyn. Among the soft Eurosceptic parties on the left, the radical left-wing Socialist Party lost five seats and the deep green Party for the Animals won one seat.

## On the European dimension

We could classify the Dutch parties along the EU dimension with five positions (Table 2; see also Vollaard and Voerman, 2015). These categories also line up with how parties are placed on the EU dimension by the experts in the Chapel Hill Expert Survey (Bakker et al., 2020). At the most pro-European side, there are two parties that advocate the EU becoming a fully fledged federation: D66 and Volt. This implies a fundamental shift in the structure and the competences of the EU. Volt is more radical in this than D66, arguing that the EU should determine policies concerning social security and economic inequality that traditionally belong to member states. D66 favoured a eurozone budget and Eurobonds long before the current crisis (Vollaard and Voerman, 2015, 166). D66 is a member of ALDE, and Volt Netherlands is the Dutch branch of the pan-European party Volt (which sits with the Greens in the European Parliament). The parties expanded their seat total from 19 to 27.

Table 2
**Parties on the EU dimension**

| Position | Parties (with CHES positions) | 2017 | 2021 |
|---|---|---|---|
| Euro-federalist | D66 (6.9), Volt (No value yet) | 19 | 27 |
| Pro-European | PvdA (5.9), GL (6.5), As1 (No value yet) | 23 | 18 |
| Euro-pragmatist | VVD (5.1), CDA (5.3), 50PLUS (3.9), CU (4.0), DENK (4.7) | 64 | 58 |
| Soft Eurosceptic | SGP (2.9), SP (2.8), PvdD (2.6), BBB (No value yet), Yes21 (No value yet) | 22 | 22 |
| Hard Eurosceptic | FVD (1.1), PVV (1.3) | 22 | 25 |

Source: Bakker et al. (2020); and author's compilation.

Then there are the pro-European parties: the Labour Party and GreenLeft as well as the new anti-racist, anti-capitalist, feminist party As1. These parties advocate strengthening solidarity in the European Union, for instance, by adopting common rules to fight tax avoidance and increasing spending at the European level. They also want to strengthen democracy in the EU and are critical of the role of big business interests. The Labour Party's EU policy has been less consistent veering between a pro-European and a more Euro-pragmatist course (Vollaard and Voerman, 2015, 137-138). With Frans Timmermans as its lead candidate for the European elections (and the subsequent reward for that choice in the elections), the PvdA has steered a more pro-European course. The GreenLeft has become more pro-European in recent decades, realising that for the eurozone to work, further economic and budgetary integration is necessary. The PvdA is a member of the Party of European Socialists, and the GreenLeft is a member of the European Greens. As a bloc, these parties lost five out of 23 seats.

Next, the Euro-pragmatist parties or self-styled "Eurorealists" (Harryvan and Van der Harst, 2013, 275) have accepted the EU as it is, but do not favour further EU integration (Kopecký and Mudde, 2002). In their view, the EU is primarily a free trade area. The common currency is there to promote free trade. These parties are reluctant to hand over more competences to the EU, for instance on taxation, and are opposed to making the eurozone a transfer union. These parties are the VVD, CDA, the pensioners' party 50PLUS, CU and the party of, for and by people with a migration background, DENK. The VVD is a member of ALDE but is more Eurosceptic than the mainstream in this group: the national (economic) interest determines their EU policy (Vollaard and Voerman 2015, 161). The CDA, 50PLUS and CU all sit (or sat) in the group of the European People's Party. Of these, the CU is least committed to the euro and has hinted that it would not lament if weaker economies left the eurozone, betraying the party's Eurosceptic past (Vollard and Voerman, 2015, 127). These parties together lost six out of 64 seats.

Additionally, there are the soft Eurosceptic parties. These parties are opposed to the current policies of the European Union and propose reducing the EU to an intergovernmental body, stripping it of its supranational features. These are the SGP, SP, the deep green PvdD, the farmers' interest party Farmer-Citizen Movement and the moderate radical right-wing populist party Yes21. SP and PvdD sit (or sat) in the United European Left-Nordic Green Left and favour EU regulation to protect workers and animals. The SGP and the Yes21 MEPs (that all left FVD) currently sit in the European Conservatives and Reformers. They, like the Euro-pragmatist parties, see the EU primarily as a free trade zone. These parties kept 22 seats combined.

Finally, there are the hard-Eurosceptic parties (FVD and PVV). These parties advocate for the Netherlands leaving the EU. For these parties, the EU has become a monstrosity that cannot be reformed. They look at the arrangements that Switzerland, Norway and now even the UK have as alternatives to EU membership (Vollaard and Voerman, 2015, 171). The PVV sits in the Identity and Democracy group, and the FVD lost its MEPs to Yes21. These parties expanded their seats from 22 to 25.

We can clearly see that the victors of the elections were at the extremes of the EU dimension. Both the most pro-European parties (D66 and Volt) and the most Eurosceptic party won (FVD) seats. The EU might not have been a major issue in the public debate surrounding the election, but the polarisation on the EU dimension is clearly visible. There are specific choices in the campaign that led to this outcome (e.g. the selection of Kaag as D66 leader and the choice of FVD to campaign on COVID-19). But from a more distant perspective, it may be that in the absence of a substantial debate between the economic left and economic right, voters may have expressed a deeper underlying cultural division between cosmopolitans and patriots in their vote. The importance of this division has grown in the past few years (De Vries, 2018; De Vries et al., 2013). Thus, the Elephant of EU politics was not absent in the minds of citizens.

### The EU policy of a new government

What does this mean for the EU policy of the Netherlands in the coming parliamentary term? For the Netherlands, the single most important EU issue is the future of the eurozone. The Netherlands has always seen EU integration primarily as an economic enterprise (Harryvan and Van der Harst, 2015). In the past eleven years, the Netherlands has argued for restrictive fiscal policies and has been a brake on transferring money to economically weaker states without restrictions. During the coronavirus pandemic, the Dutch cobbled together an informal alliance of the 'frugal four' (Sweden, Denmark, Austria and the Netherlands) to, albeit unsuccessfully, prevent debt mutualisation. The key issue in the coming term will be whether the crisis measures taken in the COVID-19 pandemic will have a lasting character. That is, whether the recovery fund with its sovereign bonds will be a permanent feature of the EU cooperation on the one hand; and whether criteria of the Stability and Growth Pact that have been waived in the crisis will return after the coronavirus crisis has faded on the other. These issues will certainly need to be addressed by the new government. The parties may have been able to ignore the EU Elephant during the three month campaign, but they can no longer ignore it over the next four years.

The elections are only one step into the Dutch political process. The votes of Dutch voters disappear in what some have called the Bermuda Triangle of Dutch politics: the process of coalition formation (Van Keken and Kuijpers, 2021). Here, the preferences of the different parties are transformed into a coalition agreement that binds the parties together. The previous coalition agreement committed the parties to a stringent eurozone course opposing future bailouts, Eurobonds, permanent economic stabilisation mechanisms and a strict application of the Stability and Growth Pact – despite the presence of the Euro-federalist D66 at the negotiating table. Its three Euro-pragmatist negotiating partners were able to determine the agenda.

Which parties will join the new government is still unclear days after the election. The formation of a new government will take some time: 94 days on average in the postwar period (Ecker and Meyer, 2015). Currently, it looks as though the Liberal Party and D66 will form the core of this new government. EU policy will mostly be determined by the interplay between VVD and D66 and their prospective government partners.

The Euro-pragmatist and fiscally conservative VVD will continue to act as a brake on anything resembling a transfer union. The liberals only accepted deviating from the rules of the Stability and Growth Pact because of the exceptional circumstances. The growth of the radical right may put electoral pressure on the VVD not to deviate from their current course. If it is up to the VVD, Rutte's reputation as "Mr. No" will continue (Van Wiel, 2020).

D66, however, will want to chart a much different course, both in tone and in policy. Certainly, D66 will want the government to take a less adversarial path regarding other EU member states. They are more open to the possibility that the crisis measures (the steps towards debt mutualisation, European taxation and a eurozone budget) will be made permanent. The entry of Volt into the parliamentary arena may also pressure them to bring their pro-European promises to fruition.

The minister of finance, together with the prime minister, are the faces of Dutch EU and euro policy: since 1989,[2] the second largest party in the government has supplied this position. This means that a more pro-European party D66 is likely to get this position and with it greater influence on EU policy.

Much will depend on which government will be formed in the extremely fractionalised landscape. Many configurations are possible, but they will need to bridge major divides regularly. The policies that the parties agree on in the coalition agreement are those that most parties desire (Willumsen and Otjes, 2019). If the current government continues, D66 will be outgunned by Euro-pragmatist parties. The same is true if VVD and D66 are joined by other less conventional partners like the soft-Eurosceptic Yes21, SGP or the SP. All these variants have a majority in the *Tweede Kamer* but will run into substantial differences on other issues like migration, moral matters and socio-economic policies. D66 has pushed for a more progressive coalition. One variant might be VVD-D66-PvdA-GL, which would add pro-European parties of the centre-left to the VVD-PvdA core government. The problem is, however, that this coalition does not have a majority in the *Tweede Kamer*, and parties will try to keep the number of coalition parties as small as possible.

2 With a brief exception in 2002-2003.

## Conclusion

The Dutch elections are, as Dutch elections often are, important but not decisive in determining the country's policies. They distribute the cards for the upcoming formation. Two parties have been dealt a good hand: the Euro-pragmatist, conservative liberal VVD and Euro-federalist, social-liberal D66. Hovering over the table, will be the EU Elephant, the inevitable question of whether the current European economic measures are merely temporary stop-gap procedures to deal with an unprecedented crisis or the start of a European transfer union. Both D66 and VVD are under electoral pressure to stay the course. Much now depends on how they play the game and, in particular, which other parties will be invited to the table. In this respect, the VVD appears to have a stronger hand because the centre-right, Euro-pragmatist parties have performed better than the left-wing pro-European parties.
